# Association between cholesterol efflux capacity and peripheral artery disease in coronary heart disease patients with and without type 2 diabetes: from the CORDIOPREV study

**DOI:** 10.1186/s12933-021-01260-3

**Published:** 2021-03-25

**Authors:** Elena M. Yubero-Serrano, Juan F. Alcalá-Diaz, Francisco M. Gutierrez-Mariscal, Antonio P. Arenas-de Larriva, Patricia J. Peña-Orihuela, Ruth Blanco-Rojo, Javier Martinez-Botas, Jose D. Torres-Peña, Pablo Perez-Martinez, Jose M. Ordovas, Javier Delgado-Lista, Diego Gómez-Coronado, Jose Lopez-Miranda

**Affiliations:** 1 Lipids and Atherosclerosis Unit. Servicio de Medicina Interna, Reina Sofia University Hospital, Maimonides Institute for Biomedical Research in Córdoba, University of Córdoba, Córdoba, Spain; 2 CIBER Physiopathology of Obesity and Nutrition (CIBEROBN), Institute of Health Carlos III, Madrid, Spain; 3 Research and Development Department, Biosearch Life, Granada, Spain; 4 Department of Biochemistry-Research, Hospital Universitario Ramón Y Cajal, Instituto Ramón Y Cajal de Investigacion Sanitaria (IRyCIS), Madrid, Spain; 5 Jean Mayer US Department of Agriculture Human Nutrition Research Center On Aging, Tufts University School of Medicine, Boston, MA USA; 6 IMDEA-Food Institute, CEI UAM + CSIC, Madrid, Spain

**Keywords:** Peripheral artery disease, Type 2 diabetes mellitus, Cholesterol efflux capacity, Coronary heart disease, Secondary prevention

## Abstract

**Background:**

Peripheral artery disease (PAD) is recognized as a significant predictor of mortality and adverse cardiovascular outcomes in patients with coronary heart disease (CHD). In fact, coexisting PAD and CHD is strongly associated with a greater coronary event recurrence compared with either one of them alone. High-density lipoprotein (HDL)-mediated cholesterol efflux capacity (CEC) is found to be inversely associated with an increased risk of incident CHD. However, this association is not established in patients with PAD in the context of secondary prevention. In this sense, our main aim was to evaluate the association between CEC and PAD in patients with CHD and whether the concurrent presence of PAD and T2DM influences this association.

**Methods:**

CHD patients (n = 1002) from the CORDIOPREV study were classified according to the presence or absence of PAD (ankle-brachial index, ABI ≤ 0.9 and ABI > 0.9 and < 1.4, respectively) and T2DM status. CEC was quantified by incubation of cholesterol-loaded THP-1 cells with the participants' apoB-depleted plasma was performed.

**Results:**

The presence of PAD determined low CEC in non-T2DM and newly-diagnosed T2DM patients. Coexisting PAD and newly-diagnosed T2DM provided and additive effect providing an impaired CEC compared to non-T2DM patients with PAD. In established T2DM patients, the presence of PAD did not determine differences in CEC, compared to those without PAD, which may be restored by glucose-lowering treatment.

**Conclusions:**

Our findings suggest an inverse relationship between CEC and PAD in CHD patients. These results support the importance of identifying underlying mechanisms of PAD, in the context of secondary prevention, that provide potential therapeutic targets, that is the case of CEC, and establishing strategies to prevent or reduce the high risk of cardiovascular events of these patients.

*Trial registration* https://clinicaltrials.gov/ct2/show/NCT00924937. Unique Identifier: NCT00924937
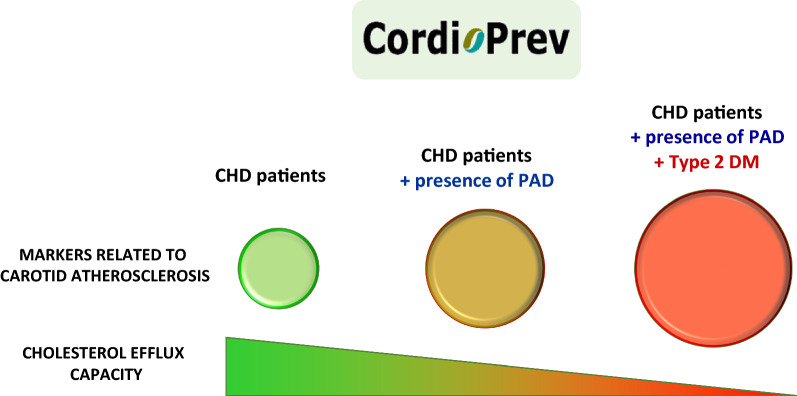

## Background

Peripheral artery disease (PAD) is characterized by the development of atherosclerotic occlusion of arteries in the lower extremities. PAD is recognized as one of the more significant predictors of mortality and adverse cardiovascular outcomes in patients with coronary heart disease (CHD) [1]. In fact, coexisting PAD and CHD is strongly associated with a greater coronary event recurrence compared with either one of them alone [2]. PAD risk factors, similar to those for CHD (such as age, hypertension, dyslipidemia or type 2 diabetes mellitus, T2DM), are also involved in its progression and are associated with a worsening of arterial perfusion of the lower extremities [3, 4].

The ankle-brachial index (ABI; the systolic blood pressure obtained at the ankle divided by the systolic blood pressure obtained at the brachial artery) is a simple, non-invasive and inexpensive tool accepted as a diagnostic test in the evaluation of PAD [5]. A low ABI value is a strong and independent predictor of fatal and nonfatal cardiovascular events and ischemic stroke [6, 7].

Reduced high-density lipoprotein (HDL)-cholesterol levels have been associated with increased risk of cardiovascular complications and mortality in PAD patients [8]. Several randomized controlled trials have failed to show a relationship between the pharmacologic improvement of HDL-cholesterol levels and a decreased risk of cardiovascular events [9, 10]. Accordingly, some clinical approaches have moved away from HDL-cholesterol levels towards HDL function as a causal risk factor for cardiovascular disease (CVD) [11]. HDL particles not only protect against atherosclerosis exerting vasodilatory effects and decreasing inflammation and oxidative stress but also by its action through the reverse cholesterol transport (RCT), considered the key cardioprotective property of HDL [12, 13]. RCT is the physiological process by which cholesterol in peripheral tissues is transported by HDL to the liver for excretion. In the initial step of RCT, through a process termed cholesterol efflux, HDLs accept cholesterol from cells, including artery-wall macrophages [14, 15]. Several studies suggest that cholesterol efflux capacity (CEC) may be a potential biomarker of atherosclerosis development, being inversely associated with the incidence of CHD, independent of HDL-cholesterol concentrations [16–18]. It is recently demonstrated that, in patients with chronic renal disease, aberrations in delivery of cholesterol effluxed from macrophages to liver may underlie the increased risk of coronary disease found in these patients [19].

However, the association between CEC and PAD is not well established. Only a recent prospective study, the MESA—Multi-Ethnic Study of Atherosclerosis, has analyzed the relationship between CEC and risk of PAD in a multi-ethnic cohort, free of baseline CVD, finding no association between CEC and risk of either clinical or incident subclinical PAD [20]. To the best of our knowledge, there are no studies evaluating the association between CEC and PAD in the context of secondary prevention.

T2DM is positively associated with macrovascular complications, being atherosclerotic CVD the leading cause of death in this population [21]. The combined presence of T2DM and CVD increases cardiovascular risk, as demonstrated by the high recurrence rate of major atherosclerotic complications (~ 6%/year) in the diabetic population [22]. Moreover, T2DM predisposes to PAD development [23]. Indeed, T2DM patients have a 2- to 4-fold greater risk of developing PAD than non-diabetic patients [24, 25]. We have recently found an inverse association between CEC and T2DM development in CHD patients, supporting the fact that cholesterol efflux could be an independent risk factor for the development of CHD and other chronic diseases, like T2DM [26].

Therefore, this study aimed to evaluate whether CEC was associated with PAD in CHD patients, with and without T2DM, for the purpose of identifying underlying mechanisms of the disease that could provide potential therapeutic target to prevent or reduce the high risk of cardiovascular events of these patients.

## Methods

### Design and study population

The current work was conducted within the framework of the CORDIOPREV (CORonary Diet Intervention with Olive oil and cardiovascular PREVention) study (Clinicaltrials.gov number NCT00924937). The CORDIOPREV study is an ongoing prospective, randomized, single-blind, controlled trial, including 1002 CHD patients who had their last coronary event more than six months before enrolment. Patients were recruited from November 2009 to February 2012, mostly at Reina Sofia University Hospital, Cordoba, Spain, and other hospitals in Cordoba and Jaen. Full details of the rationale, study methods, inclusion and exclusion criteria, cardiovascular risk factors and baseline characteristics of the patients have been described recently [27]. To summarize, patients were eligible if they were aged 20–75 years, with established CHD but without clinical events in the last six months, intending to follow a long-term monitoring study, with no other serious illnesses and a life expectancy of at least 5 years. All the patients gave their written informed consent to participate in the study. Following institutional and Good Clinical Practice guidelines, the Human Investigation Review Committee approved the study protocol at Reina Sofia University Hospital.

For the specific aims of this work, we performed a cross-sectional analysis of the CORDIOPREV population at baseline, in which the patients were classified according to 1) the presence or absence of PAD, based on their ABI value (patients with PAD, with an ABI ≤ 0.9; and patients with non-PAD, those with an ABI > 0.9 and < 1.4) [28] and 2) diabetes status (describe below). Those patients with and ABI ≥ 1.4 were excluded of the analysis.

### Diabetes status criteria

The second classification of the study patients was carried out according to their diabetic status, at baseline: non-T2DM patients, those who did not meet the criteria for T2DM diagnosis proposed by the American Diabetes Association [29], newly diagnosed T2DM patients, those who had no previous history of T2DM, being diagnosed during the recruitment period of the study and thus, without diabetic treatment; established T2DM patients, those with a prior medical history of T2DM when entering the study and that were receiving diabetic treatment.

### Laboratory tests

At 8.00 am, following a 12-h fast, the patients were admitted to the laboratory for anthropometric and biochemical tests [BMI, waist circumference, systolic blood pressure (SBP), diastolic blood pressure (DBP), HDL-cholesterol, LDL-cholesterol, triglycerides, cholesterol, high sensitive C-reactive protein (hsCRP), fasting glucose and insulin and hemoglobin A1c (HbA1c) as described previously [27]. Glomerular filtration rate (eGFR) was estimated with the Chronic Kidney Disease and Epidemiology equation (CKD-EPI) [30].

### ABI measurement

Baseline evaluation of ABI was performed according to a standardized protocol by trained examiners. BP measurements were performed with a Doppler device (Minidop ES-100X, Hadeco/Hayashi Denki Co., Ltd.) after the participants rested supine for 10 min. Brachial SBP was measured in both arms by placing the Doppler transducer above the cubital segment of both brachial arteries. For systolic ankle pressure measurement, the posterior tibial and dorsalis pedis artery was measured for each leg. ABI was calculated per leg as the higher SBP in the posterior tibial or dorsalis pedis divided by the higher of the two-arm SBPs. The lower ABI of both legs was used as the participant-specific ABI. We imputed missing data (< 7%) with Multivariate Imputation by Chained Equations (MICE) package (3.8.0) in R. All ABI measurements were performed by a single independent examiner who was unaware of other clinical data.

### Assessment of HDL-mediated cholesterol efflux capacity

Blood samples were collected, at baseline, from 12-h fasting subjects in EDTA-containing tubes, placed on ice, centrifuged at 4 °C, and stored at -80 °C. Cholesterol efflux capacity was measured at baseline as previously described [26]. Efflux assays were performed using human THP-1 monocytes (ATCC TIB-202). For the assays, THP-1 cells were plated in 48 multi-well plates at a concentration of 125,000 cells/well and they were treated with phorbol 12-myristate 13-acetate (50 ng/mL) for 72 h to become fully differentiated macrophages. Then, THP-1 macrophages were labelled with 1.2 μCi/mL [1,2-^3^H(N)]-cholesterol (Perkin-Elmer) and cholesterol loaded with 50 µg/mL of acetylated-LDL (acLDL) in RPMI medium containing 10% lipoprotein deficient fetal bovine serum (density > 1.21 g/L). After 24 h, cells were washed 3 times with phosphate buffered saline containing 0.1% human serum albumin (HSA) to remove the excess of ^3^H-cholesterol and acLDL and they were equilibrated in serum-free medium overnight at 37 °C. The next day, plasma samples, from the study patients, were thawed and treated with polyethylene glycol to precipitate apolipoprotein B-containing lipoproteins. Briefly, 40 parts polyethylene glycol solution (20% polyethylene glycol 8000 molecular weight in 200 mM glycine buffer, pH 7.4) were added to 100 parts plasma and incubated at room temperature for 20 min before spinning in a microcentrifuge at 10,000 rpm for 30 min at 4 °C. The supernatant, which contained the HDL fraction, was recovered. Finally, efflux medium containing 2% apoB-depleted plasma was added to THP-1 macrophages. The efflux period was 4 h, at 37ºC, after which the medium was removed for quantifying the ^3^H − cholesterol present therein and in cells by scintillation counting. Each sample was run in triplicate, and within each plate were always included, besides the study subjects’ plasmas, serum-free medium containing 0.2% HSA and an inter-assay control (2%) consisting of pooled apoB-depleted plasmas from four healthy volunteers and stored at − 80 °C.

We calculated the percentage efflux to the medium by the formula: (disintegrations per minute (dpm) ^3^H − cholesterol in the medium × 100)/(dpm ^3^H − cholesterol in the medium + dpm ^3^H − cholesterol in cells). The efflux to serum-free medium value in each batch was subtracted from the corresponding plasma values. To standardize the percentage efflux obtained in the several analyses, we normalized values for the study patients to the inter-assay control in each batch as follows: (study patient cholesterol efflux × 100)/inter-assay control cholesterol efflux [31]. The inter-assay variability across plates was controlled by the inter-assay control. The inter-assay coefficient of variation was 6.4% and the intra-assay coefficient of variation was 4.5%.

**Fig. 1:**
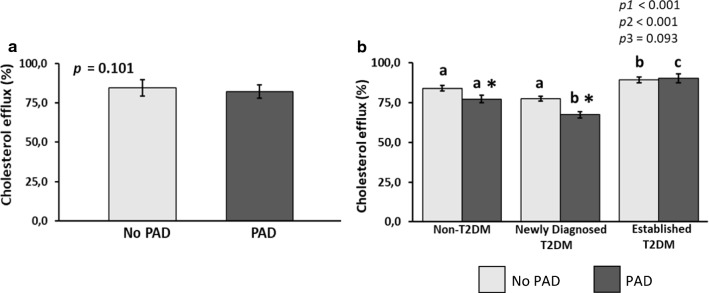
Cholesterol efflux capacity in patients with CHD. A) According to the presence or absence of PAD and B) According to the presence or absence of PAD and diabetes status * Significant differences between PAD and no PAD in each diabetes status s group. Different common letter superscripts denote significant differences among non-T2DM, newly-diagnosed T2DM and established T2DM. p1, effect of presence or absence of PAD; p2, effect of diabetes status; p3, interaction between presence of absence of PAD and diabetes status. CHD, coronary heart disease; PAD, peripheral artery disease; T2DM, type 2 diabetes mellitus Analyses were adjusted age, smoking habit, hypertension, eGFR, and medications—glucose-lowering treatment, lipid-lowering therapy, and anti-hypertensive drugs

From the total patients of the CORDIOPREV study (n = 1002), we included those whose cholesterol efflux assessment, and analytical and anthropometric data were available (n = 961). The reasons for the lack of data for the remaining 41 patients were as follows: 37 refused to undergo the ABI measurement and 4 exhibited an ABI ≥ 1.4. All participants were of European ancestry (Fig. 1).

### Statistical analysis

The statistical analyses were carried out using SPSS version 19.0 for Windows (SPSS Inc., Chicago, IL, USA). The Kolmogorov–Smirnov normality test was performed to evaluate the distribution of the quantitative variables, and continuous variables that deviated significantly from the assumption of normality were transformed. Categorical variables were compared using Chi-Square tests. Continuous data were compared using unpaired t-tests when comparing two groups or analysis of variance (ANOVA), adjusted for potential cofounders or potential effect modifiers (age, smoking habit, hypertension, eGFR, and medications—glucose-lowering treatment, lipid-lowering therapy, and anti-hypertensive drugs).

Backward multiple logistic analysis was carried out to estimate the independent contribution of diabetes status and parameters related to glucose metabolism (fasting glucose and HbA1c), cholesterol efflux capacity and different cardiovascular risk factors such age, HDL-cholesterol, triglycerides, hsCRP, eGFR, hypertension, glucose-lowering treatment and lipid-lowering therapy to the presence of PAD.

The differences were considered significant when *p* < 0.05. All the data presented in figures and tables are expressed as means ± standard error (SE).

## Results

### Study population characteristics based on the presence or absence of PAD

Of the total population, 188 CHD patients (19.6%) exhibited PAD, with an ABI ≤ 0.9 (n = 185). Table 1 shows the clinical and metabolic characteristics, lipid profiles and treatment regimens of the study patients classified according to the presence or absence of PAD. Patients with PAD were older (*p* < 0.001) and more frequently current or past smokers (*p* = 0.041) with higher levels of hsCRP (*p* < 0.001), compared to those without PAD. Patients with PAD also had lower levels of HDL-cholesterol (*p* = 0.030), higher levels of triglycerides, and greater SBP and prevalence of hypertension compared with their counterparts (all *p* < 0.01). Regarding glycemic control, PAD patients showed higher levels of fasting glucose and HbA1c, a higher prevalence of T2DM with more oral antidiabetic medication, and lower eGFR compared to non-PAD patients (all *p* < 0.05).

**Table 1:** Study population characteristics based on the presence or absence of PAD

	Total population (n = 961)	No PAD (n = 773)	PAD (n = 188)	*p* value*
Age (years)	59.6 ± 0.3	58.9 ± 0.3	62.2 ± 0.5	< 0.001
Men (%)	82.4	81.0	85.5	0.138
BMI (kg/m^2^)^a^	31.1 ± 0.1	31.1 ± 0.2	31.4 ± 0.3	0.319
Smoking (%, current or past)	10.9	9.1	13.4	0.041
Diastolic blood pressure (mmHg)	77.2 ± 0.3	77.5 ± 0.4	76.5 ± 0.8	0.252
Systolic blood pressure (mmHg)	138.8 ± 0.6	137.2 ± 0.7	145.1 ± 1.4	< 0.001
LDL-cholesterol (mg/dL)	88.5 ± 0.8	87.9 ± 1.1	88.9 ± 1.2	0.211
HDL-cholesterol (mg/dL)	42.2 ± 0.3	43.1 ± 0.4	41.2 ± 0.7	0.030
Total cholesterol (mg/dL)	159.0 ± 1.0	158.3 ± 1.1	161.9 ± 2.4	0.950
Triglycerides (mg/dL)	135.4 ± 2.2	132.3 ± 2.4	147.7 ± 5.2	0.005
hsCRP (mg/mL)	3.10 ± 0.11	2.83 ± 0.12	4.15 ± 0.30	< 0.001
Fasting glucose (mg/dL)	113.6 ± 1.2	111.7 ± 1.3	121.1 ± 2.4	0.002
Fasting insulin (mU/L)	10.9 ± 0.3	10.6 ± 0.3	12.2 ± 1.1	0.076
HbA1c (%)	6.64 ± 0.03	6.56 ± 0.04	6.99 ± 0.10	< 0.001
T2DM (%)^b^	53.8	50.1	67.1	< 0.001
eGFR (mL/min/1.73 m^2^)	89.0 ± 0.5	90.2 ± 0.6	84.0 ± 1.3	< 0.001
Hypertension (%)^c^	68.5	64.9	81.1	< 0.001
Anti-hypertensive use (%)	90.7	89.5	92.0	0.285
Lipid-lowering therapy (%)	85.7	85.0	87.6	0.379
Oral antidiabetic use (%)	34.8	31.1	50.2	< 0.001

Presence of PAD was defined as an Ankle-brachial index (ABI) ≤ 0.9

Values are represented as the mean ± standard error or percentage of participants, unless otherwise stated. We used unpaired *t* tests for quantitative variables and Chi-squared tests for categorical variables

*CHD*, cardiovascular heart disease; *PAD*, peripheral artery disease; *LDL*, low-density lipoprotein; *HDL,* high-density lipoprotein; *hsCRP*, high sensitive C-reactive protein; *HbA1c*, glycated haemoglobin; *eGFR,* estimated glomerular filtration rate

* No PAD vs. PAD

^a^Body mass index (BMI) was calculated as weight in kg divided by the square of height in m (kg/m^2^)

^b^Type 2 diabetes mellitus (T2DM) was defined as being diagnosed as diabetic before the start of the study [27], with a fasting blood glucose level ≥ 126 mg/dL on two occasions, or a 2-h plasma glucose level ≥ 200 mg/dL during a 75-g oral glucose-tolerance test, during the first procedures of the study

^c^Hypertension was defined as a systolic blood pressure ≥ 140 mm Hg, a diastolic blood pressure ≥ 90 mm Hg, or the use of antihypertensive therapy

### T2DM status and PAD

When we classified the patients according to diabetes status, established T2DM patients showed the highest PAD prevalence than non-T2DM and newly-diagnosed T2DM patients. Non-T2DM patients exhibited the lowest prevalence of PAD (*p* < 0.001) (data not shown). Moreover, the concomitant PAD and T2DM (in established T2DM patients but not in those newly-diagnosed T2DM) determine higher levels of total cholesterol and LDL-cholesterol compared with their counterparts without PAD (all *p* < 0.05) (Table 2).

**Table 2:** Study population characteristics based on the presence or absence of PAD and diabetes status

	Non-T2DM		Newly Diagnosed T2DM		Established T2DM	
	No PAD (n = 378)	PAD (n = 57)	No PAD (n = 150)	PAD (n = 35)	No PAD (n = 245)	PAD (n = 96)
Age (years)	57.1 ± 0.5^a^	61.9 ± 1.2*	60.1 ± 0.7^b^	60.1 ± 1.5	61.2 ± 0.5^b^	62.6 ± 0.8*
Men (%)	83.0	90.8	81.7	88.6	79.0	82.0
BMI (kg/m^2^)^a^	30.2 ± 0.2^a^	30.3 ± 0.5^a^	30.9 ± 0.3^a^	30.0 ± 0.7^a^	32.1 ± 0.3^b^	32.8 ± 0.4^b^
Diastolic blood pressure (mmHg)	77.8 ± 0.5	79.0 ± 1.3	77.0 ± 0.9	76.9 ± 1.9	76.1 ± 0.6	75.9 ± 1.0
Systolic blood pressure (mmHg)	133.4 ± 0.9^a^	143.8 ± 2.3	136.0 ± 1.6^a^	144.2 ± 3.3	142.9 ± 1.2^b^	144.1 ± 2.0
LDL-cholesterol (mg/dL)	91.6 ± 1.3^a^	91.3 ± 3.1^a^	91.2 ± 2.1^a^	94.2 ± 4.5^a^	80.3 ± 1.6^b^	87.8 ± 2.5*^b^
HDL-cholesterol (mg/dL)	44.7 ± 0.5^a^	43.9 ± 1.2^a^	42.0 ± 0.8^b^	41.5 ± 1.6^b^	39.3 ± 0.6^c^	38.0 ± 1.0^c^
Total cholesterol (mg/dL)	161.3 ± 1.5^a^	163.6 ± 3.9^a^	163.4 ± 2.6^a^	170.7 ± 5.4^a^	150.3 ± 1.8^b^	157.7 ± 2.9*^b^
Triglycerides (mg/dL)	119.9 ± 3.1^a^	138.6 ± 7.7*^a^	137.4 ± 5.8^ab^	162.4 ± 9.2*^b^	148.6 ± 4.8^b^	159.6 ± 7.4*^b^
hsCRP (mg/mL)	2.38 ± 0.17^a^	3.75 ± 0.41*	3.35 ± 0.34^b^	5.17 ± 0.71*	3.22 ± 0.23^b^	4.05 ± 0.36*
Fasting glucose (mg/dL)	93.4 ± 0.5^a^	93.4 ± 1.2^a^	110.1 ± 1.8^b^	107.9 ± 3.8^b^	141.8 ± 3.1^c^	143.7 ± 4.9^c^
Fasting insulin (mU/L)	8.8 ± 0.3^a^	9.6 ± 0.7^a^	10.4 ± 0.8^a^	10.7 ± 1.7^a^	12.6 ± 0.9^b^	15.9 ± 1.5^b^
HbA1c (%)	5.89 ± 0.02^a^	5.90 ± 0.04^a^	6.64 ± 0.06^b^	6.70 ± 0.13^b^	7.55 ± 0.09^c^	7.81 ± 0.13^c^
eGFR (mL/min/1.73 m^2^)	92.6 ± 0.7^a^	88.2 ± 0.6*^a^	89.8 ± 1.3^ab^	84.1 ± 2.8*^a^	86.5 ± 1.1^b^	81.4 ± 1.8*^b^
Hypertension (%)^b^	62.2^a^	84.1*	58.2^a^	80.1*	74.6^b^	83.9*
Smoking (% current or past)	8.1	12.3*	11.8	15.1*	9.3	13.0*
Anti-hypertensive use (%)	86.3	87.7	88.8	87.5	91.1	95.0
Lipid-lowering therapy (%)	85.3^a^	84.4^a^	83.9^a^	84.6^a^	91.3^b^	90.0^b^
Oral antidiabetic use (%)	0.0^a^	0.0^a^	0.0^a^	0.0^a^	100.0^b^	100.0^b^

Values are represented as the mean ± standard error or percentage of participants, unless otherwise stated

Continuous variables were analysed using analysis of variance (ANOVA) whereas categorical variables were analysed using Chi Square tests

Presence of PAD was defined as an Ankle-brachial index (ABI) ≤ 0.9

Diabetes status was defined as follow: non-T2DM, patients who did not met, at baseline, the criteria for diabetes diagnosis proposed by the American Diabetes Association^30^, Newly Diagnosed T2DM, patients who had no previous history of T2DM, thus being diagnosed during the recruitment period of the study; Established T2DM, with a prior medical history of T2DM before entering the study that were receiving treatment (medication or diet)

*CHD*, cardiovascular heart disease; *PAD*, peripheral artery disease; *LDL*, low-density lipoprotein; *HDL,* high-density lipoprotein; *hsCRP*, high sensitive C-reactive protein; *HbA1c*, glycated haemoglobin; *eGFR,* estimated glomerular filtration rate

* *p* < 0.05 No PAD vs. PAD. Significant differences according to diabetes status are presented with different common letter superscripts (*p* < 0.05)

^a^Body mass index (BMI) was calculated as weight in kg divided by the square of height in m (kg/m^2^)

^b^Hypertension was defined as a systolic blood pressure ≥ 140 mm Hg, a diastolic blood pressure ≥ 90 mm Hg, or the use of antihypertensive therapy

### Cholesterol efflux capacity according to the presence or absence of PAD and diabetes status

**Fig. 2:**
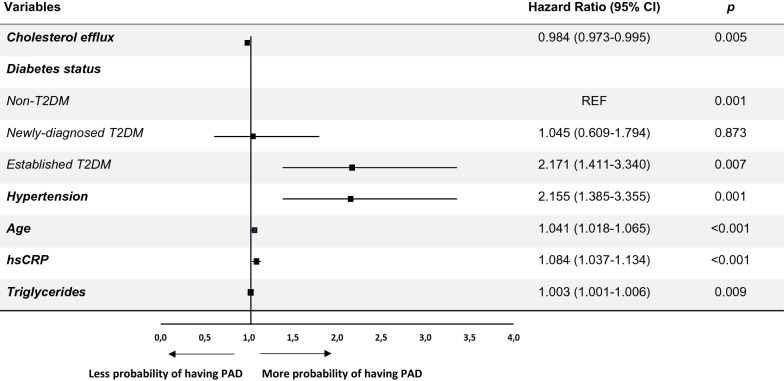
Multiple logistic regression analysis for the presence of PAD in patients with CHD. Squares denote hazard ratios; horizontal lines represent 95% confidence intervals. R^2^ = 0.201, constant = − 4.493 (p = 0.000). Predictive variables tested by backward (conditional) method: age (years), diabetes status (non-T2DM, newly-diagnosed T2DM and established T2DM), HbA1c (%), Fasting glucose (mg/dL), hypertension, HDL-cholesterol (mg/dL), Triglycerides (mg/dL), Cholesterol efflux (%), hsCRP (mg/mL), eGFR (mL/min/1.73 m^2^), oral antidiabetics use (%) and lipid-lowering therapy (%). HbA1c (%), Fasting glucose (mg/dL), HDL-cholesterol (mg/dL), eGFR (mL/min/1.73 m^2^), oral antidiabetics use (%), lipid-lowering therapy (%) have been eliminated from the model (p > 0.05). CHD, cardiovascular heart disease; T2DM, type2 diabetes mellitus; hsCRP, high sensitive C-reactive protein; eGFR, estimated glomerular filtration rate. HDL, high-density lipoprotein; HbA1c, glycated haemoglobin

In the total CHD patients, we did not find differences in CEC according to the presence or absence of PAD (Fig. 2a). However, when diabetes status was considered, both non-T2DM and newly-diagnosed T2DM patients with PAD showed lower CEC than their counterparts without PAD (*p* = 0.039 and *p* = 0.021, respectively). Moreover, in the presence of PAD, CEC was lower in newly-diagnosed T2DM compared to non-T2DM patients (*p* = 0.011). The presence of PAD, in established T2DM patients, did not determine differences in CEC compared to those without PAD (Fig. 2b).

### Multiple logistic regression model for predicting the presence of PAD

To determine the contribution of CEC to PAD, we performed a multiple logistic regression analysis (Fig. 2). Diabetes status, parameters related to glucose metabolism, and different cardiovascular risk factors were also included in the analysis. In our model, an increase of an SD of cholesterol efflux determined a decrease of 0.984-fold (95% CI 0.973–0.995) the probability of having PAD. Moreover, the presence of established T2DM (but not newly-diagnosed T2DM) had a 2.171-fold (95% CI 1.411–3.340) more likelihood of having PAD than non-T2DM (referent). Presence of hypertension (odds ratio [OR], 2.155; 95% CI 1.385–3.355), age (OR, 1.041; 95% CI 1.018–1.065), triglycerides levels (OR, 1.003; 95% CI 1.001–1.006) and hsCRP levels (OR, 1.084; 95% CI 1.037–1.134) increased the probability of having PAD.

## Discussion

Our findings are the first to evaluate CEC and PAD in patients with CHD to the best of our knowledge. In this cross-sectional study, we found an inverse relationship between CEC and PAD. The presence of PAD determined low CEC in non-T2DM and newly-diagnosed T2DM patients. Moreover, coexisting PAD and newly-diagnosed T2DM provided and additive effect providing an impaired CEC compared to non-T2DM patients with PAD. In established T2DM patients, the presence of PAD did not determine differences in CEC compared to those without PAD.

The atheroprotective properties of HDL, through its capacity to promote cellular cholesterol efflux, have been well evidenced [32, 33]. CEC of HDL is also necessary for nitric oxide activation, which enhances endothelial function (whose impairment is involved in the development of arteriosclerotic disease) by mediating anti-inflammatory and anti-oxidant activities [34]. In fact, recent findings found that reduced ABCA1 expression in the macrophages, by proprotein convertase subtilisin/kexin type 9 (PCSK9), could accelerates atherosclerosis, thereby inhibiting RCT [35, 36]. In this line, evidences have also supported that adiponectin may play a critical atheroprotective role in promoting ABCA1-dependent cholesterol efflux [37].

In this context, different studies have supported an inverse relationship between CEC and prevalence of incidence of CHD [17, 18, 33, 38]. A recent meta-analysis has described that low CEC was associated with a higher risk of CVD, even in individuals at high cardiovascular risk, irrespective of HDL cholesterol levels and other classic cardiovascular risk factors [33], suggesting that the evaluation of CEC could be a key element to understand the role of HDL in CVD.

### CEC is inversely associated with the presence of PAD in patients with CHD

Patients with advanced atherosclerosis in several territories are at very high risk of cardiovascular morbidity and mortality [1]. Hence, the importance of identifying the mechanisms involved in developing PAD in CHD patients to stratify their cardiovascular risk and apply effective therapies, as is the case of CEC. Only a clinical study has evaluated the contribution of CEC (by quantification of cholesterol mass efflux capacity, CMEC) to the risk of incident PAD in a multi-ethnic cohort, free of baseline CVD, MESA study [20]. In this prospective study, no association was found between CMEC and risk of developing either a low ABI or clinical PAD. Here, we found that an increase in cholesterol efflux reduced the probability of having PAD, suggesting that an impairment of CEC could be a mechanism involved in developing PAD in the context of established CVD, as is the case of patients with CHD. In addition to the fact that our study is carried out in the context of secondary prevention, we used a different method that one used in the MESA study. Recently, a couple of methods to evaluated CEC (a new stable isotope method and a cell-free assay) has also been developed [39, 40], whose data showed an association between CEC and the risk of coronary disease recurrence. In this sense, we could suggest that the difference in the relevance of CEC to PAD development might be attributed to the difference of CEC method.

### T2DM status is an independent contributor of the relationship between CEC and PAD

It has been reported that T2DM patients have reduced CEC [18, 41, 42], which is further decreased by CVD [42]. Our results are in line with these findings, showing that, in the presence of PAD, newly-diagnosed T2DM (without glucose-lowering treatment) exhibited lower CEC than non-T2DM patients, likely reflecting the alteration in the compositional properties of HDL. In fact, glycation of HDL, that is exacerbated by chronic hyperglycemia conditions, has been associated with loss of HDL functionality, blunting CEC in vitro [43, 44]. However, other studies have not found impaired CEC in T2DM, which may be due to oral anti-diabetic treatment [45]. In fact, in our study, we did not observe an impairment in CEC in established T2DM patients (who were under glucose-lowering treatment), regardless of PAD. In this sense, diabetes medication could act as a modulator, restoring impaired cholesterol efflux and RCT [46, 47]. It has been found that metformin increases FGF21 expression and subsequently promotes the expression of ABCA1 and ABCG1 in macrophages, promoting cholesterol efflux [48] and reducing foam cell formation [49]. Moreover, liraglutide is found to improves lipid metabolism by enhancing cholesterol efflux associated with ABCA1 and ERK1/2 pathway [50].

In our study, it should be highlighted the importance of evaluating a population of newly-diagnosed T2DM patients (without glucose-lowering therapy) since it allows to discriminate the influence of diabetes treatment and time of evolution of the disease in comparison with those patients with established T2DM.

Our study has a number of major strengths. Cholesterol efflux is the significant first step within the RCT, and the technology used is useful in several clinical and preclinical studies by us and others [26, 51, 52]. Moreover, our analysis could provide the basis for future approaches in longitudinal studies to clarify the influence of cholesterol efflux and adds insight into the molecular mechanisms involved in the development of PAD.

One of the limitations found in our study is that it is cross-sectional, which offers no evidence of causal effects. Moreover, the results are limited to CHD patients and may not be suitable for extrapolation to other populations.

## Conclusions

Our findings support an inverse relationship between CEC and PAD in patients with CHD.The concomitant presence of PAD and newly-diagnosed T2DM provided and additive effect providing an impairment in CEC. CEC was not altered in established T2DM patients (with and without PAD), which may be due to the restorative effect of CEC by glucose-lowering therapies. These results support the importance of identifying underlying mechanisms of PAD, in the context of secondary prevention, that provide potential therapeutic targets, that is the case of CEC, and establishing strategies to prevent or reduce the high risk of cardiovascular events of these patients.

## Data Availability

The data that support the findings of this study are available from the corresponding author upon reasonable request.

## Abbreviations

ABI: Ankle-brachial index
CEC: Cholesterol efflux capacity
CHD: Coronary heart disease
CORDIOPREV: CORonary Diet Intervention with Olive oil and cardiovascular PREVention
IMT-CC: Carotid intima-media thickness
PAD: Peripheral artery disease
T2DM: Type 2 diabetes mellitus
