# Correction to: Association between cholesterol efflux capacity and peripheral artery disease in coronary heart disease patients with and without type 2 diabetes: from the CORDIOPREV study

**DOI:** 10.1186/s12933-021-01269-8

**Published:** 2021-04-17

**Authors:** Elena M. Yubero-Serrano, Juan F. Alcalá-Diaz, Francisco M. Gutierrez-Mariscal, Antonio P. Arenas-de Larriva, Patricia J. Peña-Orihuela, Ruth Blanco-Rojo, Javier Martinez-Botas, Jose D. Torres-Peña, Pablo Perez-Martinez, Jose M. Ordovas, Javier Delgado-Lista, Diego Gómez-Coronado, Jose Lopez-Miranda

**Affiliations:** 1 Lipids and Atherosclerosis Unit. Servicio de Medicina Interna, Reina Sofia University Hospital, Maimonides Institute for Biomedical Research in Córdoba, University of Córdoba, Córdoba, Spain; 2 CIBER Physiopathology of Obesity and Nutrition (CIBEROBN), Institute of Health Carlos III, Madrid, Spain; 3 Research and Development Department, Biosearch Life, Granada, Spain; 4 Department of Biochemistry-Research, Hospital Universitario Ramón Y Cajal, Instituto Ramón Y Cajal de Investigacion Sanitaria (IRyCIS), Madrid, Spain; 5 Jean Mayer US Department of Agriculture Human Nutrition Research Center On Aging, Tufts University School of Medicine, Boston, MA USA; 6 IMDEA-Food Institute, CEI UAM + CSIC, Madrid, Spain

## Correction to: Cardiovasc Diabetol (2021) 20:72 https://doi.org/10.1186/s12933-021-01260-3

Following publication of the original article [1], the authors regret the error occurred in the article note for equally contribution. This has been corrected with this erratum.

The correct statement is, “Elena M. Yubero-Serrano, Juan F. Alcalá-Diaz, Diego Gómez-Coronado and Jose Lopez-Miranda contributed equally to this work” instead of “Elena M. Yubero-Serrano and Jose Lopez-Miranda contributed equally to this work”.

The original article has been corrected.
